# Beyond Titanium Carbide: The Promise of Vanadium-Based MXenes for Aqueous Supercapacitors

**DOI:** 10.3390/molecules31071097

**Published:** 2026-03-26

**Authors:** Jingyi Tan, Yi Tang, Zhao Bi, Guoqiang Dong, Miao Liu, Chenhui Yang

**Affiliations:** 1 The First Division, Institute of Hygiene of Ordance Industry, Xi’an 710065, China; 2 College of Materials Science and Engineering, Xi’an University of Science and Technology, No. 58 Yanta Road, Yanta District, Xi’an 710054, China; tangyi150@xust.edu.cn; 3 School of Chemistry and Chemical Engineering, Northwestern Polytechnical University, No. 1 Dongxiang Road, Chang’an District, Xi’an 710129, China; 4 The Seventh Division, Institute of Hygiene of Ordance Industry, Xi’an 710065, China

**Keywords:** vanadium-based MXenes, transition metal solid solution, aqueous supercapacitors, energy storage mechanisms

## Abstract

Aqueous supercapacitors are a class of crucial high-power, long-life, safe and reliable energy storage devices, with their performance fundamentally dependent on electrode materials. Two-dimensional (2D) vanadium-based MXenes, possessing rich multivalent redox activity and tunable layered structures, have emerged as one of highly promising electrode candidates, exhibiting significantly superior specific capacitance and pseudocapacitive properties compared to conventional Ti_3_C_2_T*_z_*. To overcome inherent limitations in conductivity and structural stability, this review summarizes strategies for regulating composition and microstructure through transition metal solid solution and medium-/high-entropy design. These approaches synergistically optimize electron conduction, expand ion migration pathways, and suppress electrode degradation, thereby comprehensively enhancing rate performance, cycle life, and energy density. This review systematically reveals the composition–structure–performance relationships, providing critical design insights and theoretical foundations for developing next-generation high-performance, long-life aqueous MXene-based supercapacitors.

## 1. Introduction

The global energy system is transforming profoundly. Overreliance on fossil fuels raises security and ecological concerns, making a clean-energy transition a global imperative. As major nations compete in new energy technologies, electrochemical storage emerges as a pivotal solution for grid stability and balancing supply-demand [1,2,3]. Electrochemical energy storage technology has undergone significant developmental stages, from early lead-acid batteries to contemporary lithium-ion batteries, and has now evolved into a diverse array of devices encompassing sodium-sulfur batteries, flow batteries, fuel cells, and supercapacitors. These devices generally store and release energy through redox reactions or electron transfer processes occurring at the electrode–electrolyte interface. They offer advantages such as high integration, excellent energy conversion efficiency, and environmental friendliness, yet face technical limitations including finite cycle life [4]. Among numerous electrochemical storage devices, supercapacitors stand out due to their outstanding power density, rapid response capability, and long cycle life, making them an ideal choice for high-dynamic-power scenarios. Based on their energy storage mechanisms, supercapacitors primarily comprise two types: double-layer capacitors and pseudocapacitors. Unlike lithium-ion batteries, which rely on bulk ion insertion/extraction reactions, the charge storage mechanism in supercapacitors is fundamentally based on electrostatic adsorption and rapid Faradaic processes occurring at or near the electrode surface. This effectively circumvents diffusion kinetic limitations, significantly enhancing power output performance and durability [5].

To further overcome the constraints between energy density and power performance, hybrid supercapacitors (HSCs) integrate both Faradaic and non-Faradaic charge storage mechanisms, achieving complementary performance within a single device. This has emerged as one of the frontier research directions in electrochemical energy storage [6]. The cathode of such devices typically employs double-layer capacitive materials such as porous carbon. Leveraging their high specific surface area and tunable pore structure, these materials confer high power output and extended cycle life [7,8]. The anode, conversely, utilizes pseudocapacitive or battery-type materials, significantly enhancing charge storage capacity through surface or bulk redox reactions [9,10]. Nevertheless, HSCs confront multiple technical bottlenecks: carbon-based cathodes are constrained by electrostatic adsorption mechanisms, imposing theoretical limits on specific surface area that impede further mass-specific capacity enhancement. Concurrently, highly porous structures often reduce electrode packing density, limiting volumetric energy density. Anode materials commonly suffer from poor conductivity, insufficient structural stability, and volume effects coupled with side reactions triggered by repeated ion insertion/extraction cycles, leading to progressive capacity decay. More critically, the fundamental differences between anodes and cathodes in charge storage mechanisms and reaction kinetics make it difficult to effectively match their rate performance and cycle life, constituting the core challenge constraining the comprehensive performance enhancement of HSCs [11]. Therefore, it is important to develop advanced HSCs by creating electrode materials that balance double-layer and pseudocapacitive behaviors.

2D transition metal carbides, nitrides and carbonitrides, abbreviated as MXene, constitute a novel class of 2D layered nanomaterials exhibiting graphene-like properties [12,13,14]. MXene is prepared by selectively removing A-layer atoms (Al, Si, Ga) from its precursor MAX phase (i.e., M*_n_*_+1_AX*_n_*), where M denotes early transition metals, A represents Group III-A or IV-A elements, X is C and/or N, and n = 1–4 [15,16,17]. Since the first successful synthesis of Ti_3_C_2_T*_z_* in 2011 [18], over 50 distinct MXene compositions have been synthesized, with theoretical predictions suggesting over 100 possible MXene configurations [19,20,21]. The diversity within this material family is systematically reflected across multiple dimensions, including structure, composition, and surface chemistry. Structurally, MXenes form a series of crystal structures with varying layer numbers, such as M_2_XT*_z_*, M_3_X_2_T*_z_*, M_4_X_3_T*_z_*, and M_5_X_4_T*_z_*, corresponding to different values of n [22]. Regarding elemental composition, the M site has expanded beyond the initial titanium (Ti) to include vanadium (V), molybdenum (Mo), niobium (Nb), and other transition metals, forming binary or multinary solid solutions [23]. The X site encompasses carbides, nitrides, and their hybrid forms. Furthermore, the chemical composition, coverage, and spatial distribution of surface termination groups T*_z_* (e.g., -O, -OH, -F) critically govern MXene’s physicochemical properties [24].

**Figure 1:**
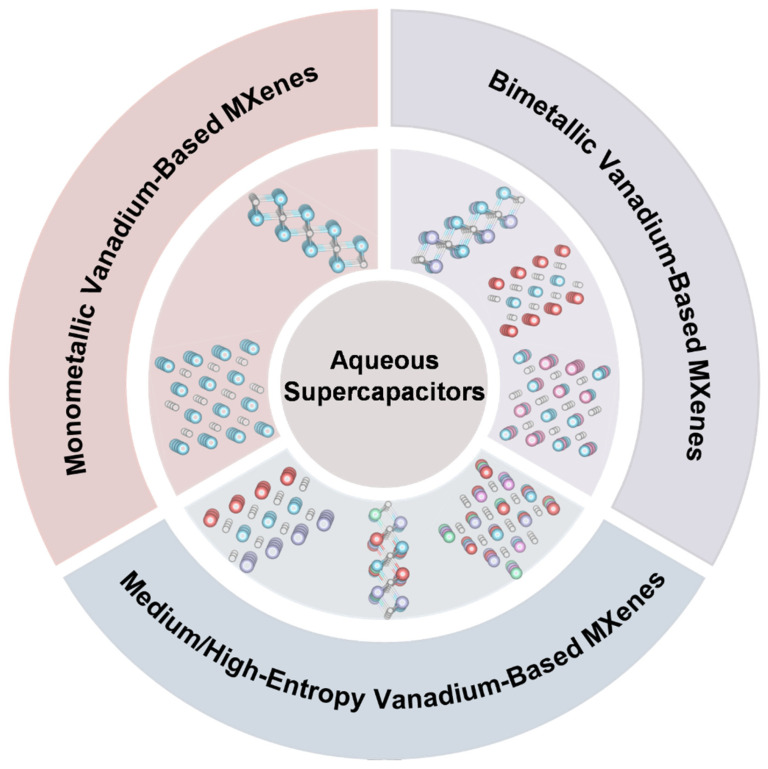
Schematic illustration of the vanadium-based MXenes structures for aqueous supercapacitors (the light blue sphere represents the element vanadium).

This high-tunability across multiple levels, including chemical composition, crystal structure, and surface chemical state, renders MXene a highly promising 2D material system for research, establishing a robust material foundation for its application in fields such as energy storage. As shown in Figure 1, unlike previous MXene reviews that broadly cover the entire family of 2D transition metal carbides and nitrides or focus predominantly on Ti_3_C_2_T*_z_*, this work provides a focused and systematic analysis of vanadium-based MXenes—a subclass distinguished by rich multivalent redox activity and exceptional pseudocapacitive behavior. Moving beyond fragmented descriptions of compositional engineering, we establish an integrated framework spanning monometallic, bimetallic, and medium-/high-entropy vanadium-based systems, elucidating coherent composition–structure–performance relationships. Particular emphasis is placed on the synergistic effects of multi-metal solid-solution and entropy-stabilization strategies, offering critical insights into overcoming the intrinsic limitations of conventional MXenes. Accordingly, this review aims to provide theoretical guidance and a dedicated roadmap for the rational design of high-performance vanadium-based MXene electrodes for aqueous supercapacitors.

## 2. Monometallic Vanadium-Based MXenes for Aqueous Supercapacitors

**Figure 2:**
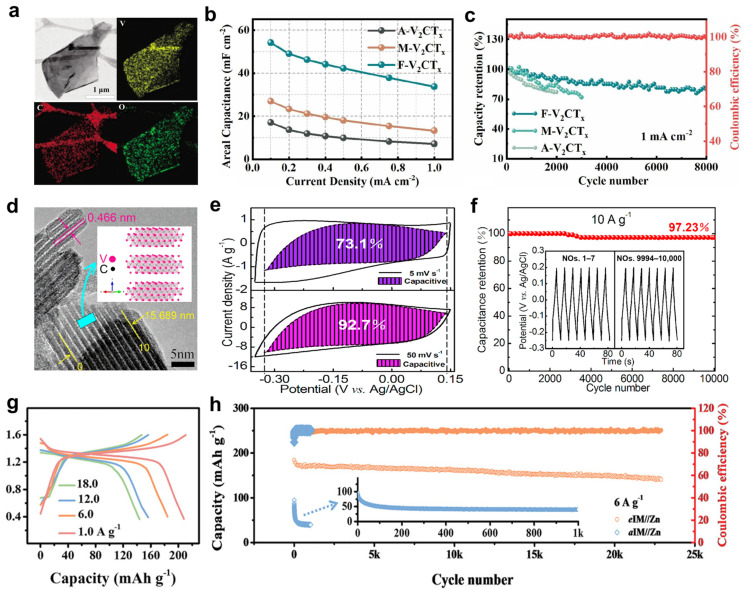
(**a**) TEM image and elemental mapping of few-layer V_2_CT*_z_* [29]. (**b**,**c**) Area capacitance comparison and cycling stability test of V_2_CT*_z_* [29]. (**d**) TEM image of V_4_C_3_T*_z_* [30]. (**e**,**f**) Pseudocapacitive contribution analysis and cycling stability test of V_2_CT*_z_* [30]. (**g**) GCD curves of Nb_2_CT*_z_* at different current densities [31]. (**h**) Cycling stability test of Nb_2_CT*_z_* [31].

MXene has garnered significant attention in the field of energy storage due to its metallic-level conductivity, open layered structure, flexible surface chemical tunability, and high specific surface area. It is particularly regarded as one of the most promising electrode materials for aqueous supercapacitors [25]. However, conventional Ti_3_C_2_T*_z_* is constrained by factors such as its relatively low theoretical capacity and narrow electrochemical window, limiting its application in supercapacitors [26,27,28]. In contrast, vanadium-based MXenes possess abundant multivalent redox states, endowing them with both high specific capacity and outstanding pseudocapacitive properties, making them highly sought-after as electrode materials for aqueous supercapacitors. Zhao et al. [29] prepared flexible electrodes from few-layer V_2_CT*_z_* via electrophoretic deposition, where the abundance of active sites and shortened ion transport pathways collectively enhanced electrochemical performance. Flexible zinc-ion hybrid capacitors assembled with this cathode achieved an area capacitance of 54.12 mF cm^−2^ at 0.1 mA cm^−2^, with a capacity retention rate of 81.48% after 8000 cycles and a self-discharge rate of merely 6.4 mV h^−2^ (Figure 2a–c).

However, the long-term cycling stability of 2D V_2_CT*_z_* materials remains somewhat limited. In contrast, V_4_C_3_T*_z_* synthesized by regulating the precursor-to-vanadium-carbon ratio exhibits superior comprehensive performance. For instance, Wang et al. [30] successfully fabricated multilayer V_4_C_3_T*_z_* by selectively etching the aluminum layer from the V_4_AlC_3_ precursor. This material achieved a specific capacitance of 209 F g^−1^ at a scan rate of 2 mV s^−1^, with its high capacitance attributed to the multivalent redox states of vanadium (V^2+^~V^4+^), an interlayer spacing of approximately 0.466 nm, a high specific surface area of 31.35 m^2^ g^−1^, and favorable surface hydrophilicity. Pseudocapacitive contributions accounted for approximately 37% of its total capacitance (268.5 F g^−1^). Notably, this electrode exhibits outstanding cycling stability with a capacity retention of 97.23% after 10,000 cycles at 10 A g^−1^, primarily due to its high electronic conductivity of 1137 S m^−1^ (Figure 2d–f). Nb-based MXenes, belonging to the same group as V, also exhibit multivalence redox properties, demonstrating favorable rate performance and cycling stability in ionic capacitors. Li et al. [31] demonstrated that Nb_2_CT*_z_*, owing to its open layered structure and high conductivity, serves as an efficient zinc storage platform. Electrodes constructed from this material exhibit a stable discharge plateau around 1.3 V, contributing 84.5% of capacity and 89.1% of energy density, alongside outstanding rate performance (143 mAh g^−1^ at 18 A g^−1^) and cycling stability (excellent capacity retention after 23,000 cycles with a self-discharge rate of merely 5.6 mV h^−1^) (Figure 2g,h). Its superior electrochemical performance is primarily attributed to the localized electron transfer and confinement effect inherent in the Nb_2_CT*_z_* matrix. This effect significantly enhances electrode reaction kinetics while effectively suppressing active material migration during cycling. Similarly, Zhao et al. [32] reported Nb_4_C_3_T*_z_* with an ultra-large interlayer spacing (approximately 1.77 nm). This structure provides ample space for reversible insertion/extraction of various cations, effectively alleviating volumetric strain during cycling. Consequently, it achieves high volumetric capacitance and favorable rate performance. Moreover, Ta-based MXenes are increasingly demonstrating significant application potential in energy storage due to their favorable comprehensive electrochemical properties and outstanding structural stability. Syamsai et al. [33] successfully prepared 2D Ta_4_C_3_T*_z_* electrode material via a solid-state approach combining hydrofluoric acid etching with ball milling-hot pressing. In a 0.1 M H_2_SO_4_ electrolyte, this material exhibited a high specific capacitance of 481 F g^−1^ at a scan rate of 5 mV s^−1^, maintaining an 89% capacity retention after 2000 cycles.

Despite the promising pseudocapacitive properties of monometallic vanadium-based MXenes, several intrinsic limitations persist. First, the relatively low electronic conductivity of V_2_CT*_z_* compared to Ti_3_C_2_T*_z_* restricts its rate capability at high current densities. Second, the long-term structural stability of V_2_CT*_z_* remains inferior to that of higher-order MXenes such as V_4_C_4_T*_z_*, highlighting the influence of layer number on durability. Third, the synthesis of pure V_4_C_3_T*_z_* phase requires precise control of the precursor stoichiometry, posing challenges for reproducible large-scale production. These trade-offs between capacitance, conductivity, and stability underscore the need for compositional and structural optimization, which forms the basis for the bimetallic and entropy-driven strategies discussed in subsequent sections.

## 3. Bimetallic Vanadium-Based MXenes for Aqueous Supercapacitors

The M sites in MXenes can accommodate various transition metal elements, forming composition-tunable solid solution structures that enable precise control over the material’s electronic properties and surface chemistry. This flexible compositional design strategy significantly broadens MXene’s application potential in electrochemical energy storage devices such as supercapacitors.

For the M_2_X configuration of bimetallic V-based MXenes, our previous work [12] synthesized V-doped Mo_2_CT*_z_* solid-solution MXenes ((Mo_1/2_V_1/2_)_2_CT*_z_* and (Mo_1/3_V_2/3_)_2_CT*_z_*) via a two-step molten salt strategy. The V incorporation introduces multivalent redox centers, enhancing pseudocapacitance and charge transfer kinetics. As a result, the optimized (Mo_1/3_V_2/3_)_2_CT*_z_* electrode delivers a high capacitance of 608.6 F g^−1^ at 0.2 A g^−1^, outstanding cycling stability (94.4% after 13,000 cycles), and an energy density of 91.8 Wh kg^−1^—far exceeding that of pristine Mo_2_CT*_z_* and most reported MXene-based Zn-ion hybrid supercapacitors.

**Figure 3:**
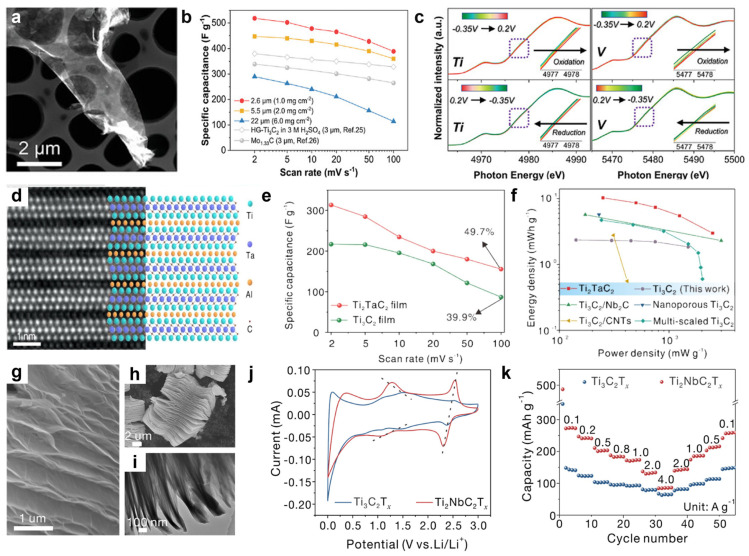
(**a**) Low-magnification HAADF-STEM image of Ti_2_VC_2_T*_z_* [34]. (**b**) Comparison of electrochemical properties for Ti_2_VC_2_T*_z_* [34]. (**c**) Ti K-edge and V K-edge XANES spectra of Ti_2_VC_2_T*_z_* at different potentials [34]. (**d**) HRSTEM image of Ti_2_TaC_2_T*_z_* [36]. (**e**,**f**) Comparison of electrochemical properties for Ti_2_TaC_2_T*_z_* [36]. (**g**–**i**) SEM image of Ti_2_NbC_2_T*_z_* [37]. (**j**,**k**) Comparison of electrochemical properties of Ti_2_NbC_2_T*_z_* [37].

For the M_3_X_2_ configuration of bimetallic V-based MXenes, as illustrated in Figure 3a–c, He et al. [34] successfully sintered the solid-solution MAX phase Ti_2_VAlC_2_ by introducing the highly redox-active element V into the Ti_3_AlC_2_ phase. Subsequently, Ti_2_VC_2_T*_z_* was obtained via HCl and KF etching. This material exhibits outstanding electrochemical performance in acidic electrolytes, achieving a specific capacity of 520 F g^−1^ with a capacity retention exceeding 90% after 20,000 cycles. Additionally, the Ti_2_VC_2_T*_z_* prepared in our previous work [35] exhibits a high volumetric capacity of approximately 1800 F cm^−3^ (5 mV s^−1^) in a 1 M H_2_SO_4_ electrolyte—significantly outperforming typical Ti_3_C_2_T*_z_* and V_2_CT*_z_* systems. Research indicates that its energy storage mechanism stems from the synergistic interaction between surface redox reactions driven by Ti/V multivalence changes and hydrated hydrogen ion intercalation, wherein the incorporation of V significantly enhances the material’s redox pseudocapacitive response.

To investigate the universal principles governing solid-solution elements, further studies were extended to multiple metallic elements beyond V. This systematically revealed the regulatory mechanisms of different metallic solid solutions on the microstructure and capacitive properties of MXene. As depicted in Figure 3d–f, Guan et al. [36] achieved the first successful synthesis of a bilayer transition metal MXene (Ti_2_TaC_2_T*_z_*) featuring an out-of-plane ordered structure, wherein Ti and Ta atoms occupy the outer and intermediate metal layers respectively. Theoretical calculations indicate that the incorporation of Ta significantly enhances the material’s work function and shifts the *d*-band center closer to the Fermi level, thereby optimizing its electronic structure. This material exhibits outstanding electrochemical performance: a specific capacitance as high as 313 F g^−1^, no capacity decay after 10,000 cycles at a current density of 10 A g^−1^, and an energy density four times that of previous Ti_3_C_2_T*_z_*-based symmetric supercapacitors. The performance enhancement is primarily attributed to Ta’s expanded operating potential window, higher density of d orbital states, and controllable surface chemistry.

Building upon research in the Ti-Ta system, Guan et al. [37] further extended the applicability of the M-site solid solution strategy by introducing Nb into the M-site of Ti_3_C_2_T*_z_*. This led to the successful synthesis of a double transition metal MXene (Ti_2_NbC_2_T*_z_*) featuring an out-of-plane ordered structure. Research indicates that, compared to Ti_3_C_2_T*_z_*, Nb alloying not only increases the interlayer spacing of Ti_2_NbC_2_T*_z_*, thereby significantly enhancing Li^+^ diffusion rates, but also reduces charge transfer resistance. This material exhibits a reversible capacity of up to 272 mAh g^−1^ at a current density of 0.1 A g^−1^, maintaining capacity stability after 1000 cycles and demonstrating electrochemical performance far surpassing that of Ti_3_C_2_T*_z_* (Figure 3g–k). Beyond the titanium-based framework, MXenes incorporating other transition metals such as vanadium and molybdenum as primary solid-solute components have been successfully developed, further expanding the compositional space and performance tunability of this material system. Our previous work [38] successfully fabricated flexible, self-supporting Mo_2_VC_2_T*_z_* films dominated by oxygen-terminated sites through in situ solid-solution sintering and liquid-phase etching. This material achieves out-of-plane ordered structure within the lattice by exploiting the difference in atomic radii between Mo and V, along with V’s unique electronic configuration. Strong Mo-V d orbital hybridization and bimetallic electronic synergy optimize the density of states distribution near the Fermi level, thereby enhancing its electronic conductivity to 258.4 S cm^−1^. Concurrently, the ordered solid solution induces expanded interlayer spacing, significantly facilitating rapid ion insertion/extraction and endowing the material with outstanding multi-ion storage capabilities. Additionally, the Mo_2_VC_2_T*_z_* electrode prepared in our previous work [35] exhibited a volumetric capacitance exceeding 1000 F cm^−3^ in 1 M H_2_SO_4_, further validating the synergistic effect of its optimized electronic structure and interlayer spacing in enhancing charge storage kinetics.

Furthermore, for the M_4_X_3_ configuration of bimetallic V-based MXenes, Wang et al. [39] discovered that partially substituting V sites with Cr in V_4_C_3_T*_z_* simultaneously increases Zn^2+^ adsorption sites, expands the MXene (V_3_CrC_3_T*_z_*) lattice, and reduces the Zn^2+^ diffusion energy barrier. This significantly enhances the material’s ion transport kinetics and structural stability. Zn//V_3_CrC_3_T*_z_* hybrid supercapacitors assembled from this material achieved a specific capacitance of 397.5 F g^−1^ at 0.2 mA cm^−2^, a 38% improvement over Zn//V_4_C_3_T*_z_*, with a discharge voltage rising to 1.30 V. After 50,000 cycles, they retained 70.2% of their initial capacity, substantially outperforming the unmodified system.

While bimetallic solid-solution MXenes exhibit superior electrochemical performance compared to their monometallic counterparts, critical challenges remain. The incorporation of secondary metals often complicates the synthesis process, requiring carefully controlled etching conditions to preserve phase purity. Moreover, the distribution of metal atoms—whether ordered or disordered—significantly influences electronic properties, yet achieving precise atomic-scale control remains difficult. Additionally, the long-term stability of bimetallic MXenes in aqueous electrolytes, particularly under repeated cycling, is not yet fully understood, with limited in situ characterization studies available. Addressing these issues will be essential for translating laboratory-scale performance into practical applications.

## 4. Medium-/High-Entropy Vanadium-Based MXenes for Aqueous Supercapacitors

As an extension of the multi-component solid solution strategy in 2D materials, medium-/high-entropy MXenes, are the most extensively studied class of multimetallic MXenes, driving a paradigm shift in the field of electrochemical energy storage.

**Figure 4:**
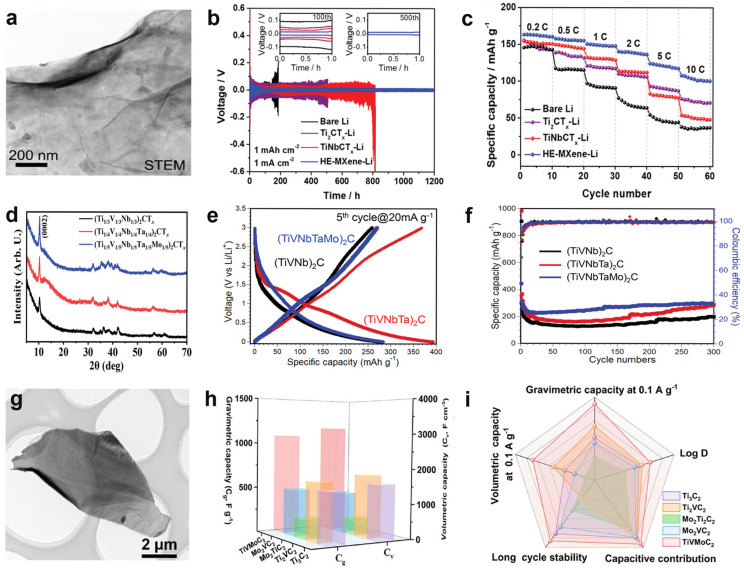
(**a**) STEM image [40]. (**b**) Galvanostatic cycling performance of symmetric cells with (Ti_1/5_V_1/5_Zr_1/5_Nb_1/5_Ta_1/5_)_2_CT*_z_*-Li symmetric cell [40]. (**c**) Rate performance of the (Ti_1/5_V_1/5_Zr_1/5_Nb_1/5_Ta_1/5_)_2_CT*_z_*-Li/LFP full cell at 0.2–10 C [40]. (**d**) XRD patterns, (**e**) Galvanostatic charge–discharge curves and (**f**) cycling stability tests for (Ti_1/3_V_1/3_Nb_1/3_)_2_CT*_z_*, (Ti_1/4_V_1/4_Nb_1/4_Ta_1/4_)_2_CT*_z_*, and (Ti_1/5_V_1/5_Nb_1/5_Ta_1/5_Mo_1/5_)_2_CT_z_ [23]. (**g**) TEM image of TiVMoC_2_T*_z_* nanosheets [35]. (**h**,**i**) Comparison of electrochemical performance among five electrodes including TiVMoC_2_T*_z_* [35].

For the M_2_X configuration of medium-/high-entropy V-based MXenes, Du et al. [40] successfully synthesized (Ti_1/5_V_1/5_Zr_1/5_Nb_1/5_Ta_1/5_)_2_CT*_z_* with a uniform monolayer structure by selectively etching the high-entropy MAX phase (Ti_1/5_V_1/5_Zr_1/5_Nb_1/5_Ta_1/5_)_2_AlC. The solid solution of five transition metals and high molar configurational entropy endow the material with outstanding structural stability and significant lattice distortion. The resulting high mechanical strain energy effectively regulates lithium nucleation and growth, achieving dendrite-free deposition. Electrochemical testing demonstrates that the high-entropy MXene based lithium metal anode exhibits cycling stability for 1200 h at a capacity of 20 mAh cm^−2^, highlighting high-entropy MXene’s unique advantage in suppressing lithium dendrite formation. The assembled high-entropy MXene-Li/LFP full cell exhibits a discharge specific capacity of 170 mAh g^−1^ at 0.5 C, maintaining 150 mAh g^−1^ after 50 cycles, significantly outperforming TiNbCT*_z_* (130 mAh g^−1^) and Ti_2_CT*_z_* (135 mAh g^−1^) full cell systems (Figure 4a–c). Subsequently, Chen et al. [23] successfully synthesized three multi-component, medium-/high-entropy MXenes (Ti_1/3_V_1/3_Nb_1/3_)_2_CT*_z_*, (Ti_1/4_V_1/4_Nb_1/4_Ta_1/4_)_2_CT*_z_*, and (Ti_1/5_V_1/5_Nb_1/5_Ta_1/5_Mo_1/5_)_2_CT*_z_* under fluorine-free conditions by etching gallium-containing medium-entropy MAX and high-entropy MAX phase precursors with a Lewis acid molten salt (CuCl_2_). The resulting materials exhibit structural features characterized by multi-element random occupation of M sites and surfaces predominantly terminated with -O and -Cl groups. Electrochemical testing revealed outstanding ionic storage performance across this series: at 200 mA g^−1^, (Ti_1/4_V_1/4_Nb_1/4_Ta_1/4_)_2_CT*_z_* exhibited a discharge specific capacity approaching 400 mAh g^−1^; while (Ti_1/5_V_1/5_Nb_1/5_Ta_1/5_Mo_1/5_)_2_CT*_z_* maintained a specific capacity of 302 mAh g^−1^ after 300 cycles at 200 mA g^−1^, demonstrating good cycling stability even at high current densities (Figure 4d–f).

Furthermore, for the M_3_X_2_ configuration of medium-entropy V-based MXenes, our previous work [35] constructed medium-entropy TiVMoC_2_T*_z_* nanostructures by doping Mo and V elements into the Ti_3_C_2_T*_z_* framework, achieving simultaneous enhancement of structural robustness and electrochemical activity. The Mo element strengthened the covalent nature of M-C bonds and enhanced oxidation resistance, while the multivalent redox centers introduced by V significantly boosted pseudocapacitance. Studies indicate that the TiVMoC_2_T*_z_* electrode exhibits outstanding mass-specific capacitance (1081.6 F g^−1^) and volume-specific capacitance (3125.0 F cm^−3^) at 5 mV s^−1^, maintaining a cycle retention rate of 90.8% after 30,000 cycles, demonstrating exceptional electrochemical stability. In situ and ex situ electrochemical testing alongside theoretical analysis confirm that TiVMoC_2_T*_z_* exhibits reversible proton storage characteristics, pseudocapacitive behavior, outstanding structural stability, and a low ionic diffusion barrier (Figure 4g–i).

For the M_4_X_3_ configuration of medium-/high-entropy V-based MXenes, Etman et al. [41] successfully prepared high-entropy MXene Ti_1.1_V_0.7_Cr*_x_*Nb_1.0_Ta_0.6_C_3_T*_z_* freestanding films via selective etching. When employed as electrodes for Zn-ion hybrid supercapacitors, this material delivers a capacity of 77 mAh g^−1^ at 0.5 A g^−1^ with an 87% capacity retention after 10,000 cycles—demonstrating stable performance in both Zn(CF_3_SO_3_)_2_ and low-cost ZnCl_2_ electrolytes. Tan et al. [42] successfully synthesized a medium-entropy MXene, Ti_1.1_V_1.1_Cr_0.4_Nb_1.4_C_3_T*_z_*, by selectively etching the Al layer from a DFT-predicted TiVCrNbAlC_3_ precursor. The highly diverse composition induces lattice distortions, creating increased active sites and enhanced electrical conductivity. As a result, the annealed ME-MXene electrode delivers specific capacitances of 292.74 F g^−1^ at 2 mV s^−1^ and 137.20 F g^−1^ at 200 mV s^−1^—significantly outperforming single transition metal MXene systems.

Medium-/high-entropy MXenes represent a paradigm shift in compositional design, yet their development is still in its infancy. The major challenges include the limited availability of high-entropy MAX phase precursors, the complexity of achieving homogeneous elemental distribution, and the difficulty in controlling surface termination chemistry in multimetallic systems. Furthermore, the high configurational entropy does not universally guarantee enhanced performance; careful optimization of elemental combinations is required to avoid undesirable phase segregation or compromised conductivity. Future efforts must focus on establishing synthesis–structure–property correlations for entropy-stabilized MXenes to fully harness their potential.

## 5. Conclusions and Outlook

### 5.1. Conclusions

This review systematically elucidates how multi-metal site solid-solution engineering strategies—encompassing monometallic, bimetallic, and medium-/high-entropy V-group-based MXenes—can synergistically regulate the electronic structure, ion transport, and interfacial chemistry of 2D materials. This approach overcomes the performance limitations of conventional titanium-based MXenes in aqueous supercapacitors. By establishing a clear “composition design-structure regulation-performance output” correlation, it provides a systematic material design roadmap for developing next-generation high-performance MXene-based energy storage devices. This work emphasizes that multimetallic synergy is not merely a material modification technique but a paradigm-shifting strategy to unlock the theoretical energy storage potential of 2D transition metal carbides/nitrides. Key research findings are summarized as follows:

#### 5.1.1. Fundamental Principles of Material Design

The review demonstrates that introducing multivalent transition metals (e.g., V, Mo, Nb, Ta) into MXene’s M sites via solid solution and entropy engineering can directly optimize electrochemical performance. These strategies synergistically enhance electronic conduction (e.g., Mo-V d-orbital hybridization boosting conductivity to 258.4 S cm^−1^), expand ionic migration pathways (e.g., Nb alloying increasing interlayer spacing), and markedly improve structural stability and cycling life through lattice distortion and high configurational entropy.

#### 5.1.2. Comparative Performance of Vanadium-Based MXenes

As shown in Table 1, monometallic V_2_CT*_z_* flexible electrodes achieve an area capacitance of 54.12 mF cm^−2^ in zinc-ion hybrid capacitors with 81.48% capacity retention after 8000 cycles; V_4_C_3_T*_z_*, owing to its abundant multivalent redox states (V^2+^/V^4+^), exhibits a pseudocapacitive contribution ratio as high as 37% and a capacity retention rate of 97.23% after 10,000 cycles; Bimetallic Ti_2_VCT*_z_* achieves a specific capacity of 520 F g^−1^ in acidic electrolytes, with a capacity retention exceeding 90% after 20,000 cycles, significantly outperforming Ti_3_C_2_T*_z_* and V_2_CT*_z_* systems. Medium-/high-entropy strategies further push the performance boundaries of vanadium-based MXenes: (Ti_1/5_V_1/5_Zr_1/5_Nb_1/5_Ta_1/5_)_2_CT*_z_* achieves 1200 h of dendrite-free lithium deposition via high-entropy-induced lattice distortion; (Ti_1/4_V_1/4_Nb_1/4_Ta_1/4_)_2_CT*_z_* exhibits a lithium storage capacity approaching 400 mAh g^−1^; TiVMoC_2_T*_z_* achieves a high specific capacity of 1081.6 F g^−1^ and a capacity retention rate of 90.8% after 30,000 cycles through Mo-V synergistic effects.

#### 5.1.3. Structure-Property Correlation

The electrochemical performance of vanadium-based MXenes is governed by a complex interplay among composition, crystal structure, and surface chemistry. Introducing multivalent transition metals into the M site modulates the electronic density of states near the Fermi level via d-band center shifts and enhanced metal–carbon covalency, reducing charge transfer resistance in bimetallic and high-entropy systems. Ordered out-of-plane bimetallic structures further optimize electronic structure, achieving zero decay after 10,000 cycles. Ion transport critically depends on interlayer spacing and surface termination; expanded interlayer channels and oxygen-terminated surfaces facilitate rapid intercalation of protons and multivalent ions—such as the reversible proton storage in TiVMoC_2_T*_z_*—substantially enhancing rate capability and capacity. Redox behavior is dominated by multivalent V centers, with additional redox couples in bimetallic systems broadening the electrochemical activity window, where pseudocapacitive contributions can exceed 37% in optimized systems. The entropy-stabilization effect, arising from high configurational entropy and lattice distortion in medium-/high-entropy MXenes, effectively suppresses electrode degradation by accommodating lattice strain, thereby preserving structural integrity over tens of thousands of cycles and enabling exceptional long-term cycling stability.

#### 5.1.4. Industrial Readiness and Comparison with Non-MXene Benchmark Materials

Bimetallic vanadium-based MXenes (e.g., Ti_2_VCT*_z_*, Mo_2_VCT*_z_*) demonstrate strong potential for industrial translation, combining high specific capacitance (>500 F g^−1^ in acidic media), excellent cycling stability (>90% retention over 10,000–20,000 cycles), and scalability via HF-free etching and roll-to-roll processing. Unlike conventional non-MXene electrodes—where activated carbon offers long life but limited capacitance (<300 F g^−1^), and metal oxides or conducting polymers degrade within 10,000 cycles—vanadium-based MXenes, particularly in bimetallic and medium-entropy forms, uniquely achieve high rate capability, ultra-long cycle life (>30,000 cycles), and scalable processability. Key challenges remain in material consistency, surface termination control, and synthesis cost reduction, with advances in fluorine-free etching, continuous processing, and electrode engineering essential for bridging laboratory performance and industrial deployment.

### 5.2. Outlook

Based on this systematic review of the application progress of vanadium-based MXenes in aqueous supercapacitors, future research should advance synergistically across multiple dimensions to fully unlock their theoretical potential and accelerate practical implementation:

#### 5.2.1. Machine Learning-Assisted Material Discovery and Performance Prediction

Leverage high-throughput computing and artificial intelligence to accelerate the discovery of novel vanadium-based MXenes and their optimal solid-solution/entropy-gain combinations. Precisely predict their electronic structures, ionic diffusion energy barriers, and theoretical specific capacitance to guide efficient “composition–structure–performance” design and screening.

#### 5.2.2. Multi-Scale Hierarchical Structure Construction

At the atomic scale, design MXenes featuring multi-site ordered/disordered solid solutions, heteroatomic doping (e.g., N, B) at X sites, and precisely controlled surface terminations to synergistically optimize activity and stability. At the electrode scale, integrate 3D printing and cryogenic casting technologies to construct gradient pore channels or vertically oriented macroscopic bulk structures. This overcomes ion transport bottlenecks caused by nanoplate stacking while unifying high areal capacity with superior mechanical flexibility.

#### 5.2.3. Adaptive Electrolyte Engineering

The electrochemical performance of vanadium-based MXenes is highly dependent on electrolyte composition, as charge storage involves both surface redox reactions and ion intercalation. In acidic electrolytes (e.g., 1 M H_2_SO_4_), proton intercalation dominates, yielding high pseudocapacitance but a limited voltage window (~1.0 V). In neutral electrolytes (e.g., ZnSO_4_), Zn^2+^ intercalation enables higher operating voltages and compatibility with zinc-ion hybrid supercapacitors, albeit with reduced rate capability due to the larger ionic radius. Emerging electrolyte engineering strategies, such as water-in-salt and deep eutectic solvents, can expand the stability window beyond 2.0 V while retaining high conductivity. Anion selection also plays a critical role: triflate-based electrolytes (e.g., Zn(CF_3_SO_3_)_2_) improve cycling stability via stable solid-electrolyte interphase formation, whereas chloride-based electrolytes offer cost benefits but may promote corrosion. Future efforts should prioritize systematic correlations between electrolyte chemistry (cation size, anion type, concentration) and MXene surface termination, interlayer spacing, and long-term stability to enable rational electrolyte design tailored for specific vanadium-based MXene systems.

#### 5.2.4. Multifunctional Integrated Device Development

Leveraging the superior conductivity, flexibility, and multi-ion storage capacity of vanadium-based MXenes, develop integrated flexible micro-supercapacitor-sensor systems for wearable electronics. This enables synergistic functions such as energy storage, sensing, and electromagnetic shielding, expanding their integrated applications in self-powered systems.

#### 5.2.5. Green Sustainable Preparation and Recycling

Develop fluorine-free/low-toxicity etching processes (e.g., Lewis acid molten salt etching, electrochemical stripping) and scalable fabrication techniques. Simultaneously, explore efficient closed-loop recycling and regeneration strategies for MXene waste to reduce the full-lifecycle environmental footprint and meet industrial sustainability requirements.

#### 5.2.6. Key Challenges and Future Research Priorities

Vanadium-based MXenes demonstrate superior pseudocapacitance, with certain systems (e.g., V_4_C_3_T*_z_*, Ti_2_VCT*_z_*, and entropy-stabilized compositions) achieving capacity retention exceeding 90% over 20,000–30,000 cycles—surpassing conventional Ti_3_C_2_T*_z_* (90–95% after 10,000 cycles) and far outperforming non-MXene materials like MnO_2_ or conducting polymers, which typically degrade within 5000–10,000 cycles. Despite these advances, further improvements in long-term cycling stability under practical conditions hinge on addressing key challenges:

Stability in Multivalent Electrolytes: While Zn^2+^ and H^+^ have been studied, the cycling stability of vanadium-based MXenes in multivalent systems (e.g., Mg^2+^, Al^3+^) remains underexplored, necessitating targeted studies on compatibility and reversible insertion behavior. Degradation Mechanisms under Realistic Operation: Systematic investigation into structural evolution, interfacial side reactions, and termination-group dynamics during extended cycling is essential to identify failure modes beyond idealized aqueous systems. Scalable Synthesis with Controlled Uniformity: Achieving consistent electrochemical stability requires precise control over composition, defect density, and layer uniformity in large-scale manufacturing processes, which currently poses a critical barrier to industrial application.

#### 5.2.7. Strategic Considerations: Compositional Complexity vs. Practicality

A key strategic consideration is whether to pursue compositional complexity (e.g., medium-/high-entropy or multimetallic solid solutions) or optimize simpler single- or double-metal systems. Single-metal V-MXenes offer well-defined pseudocapacitance and facile synthesis but face limitations in conductivity and long-term stability. Bimetallic solid solutions strike a balance, enhancing rate capability and cycle life via synergistic electronic effects. Medium-/high-entropy MXenes leverage lattice distortion and configurational entropy to achieve exceptional durability, albeit with increased synthesis complexity. For ultra-long cycle life, entropy-stabilized systems are promising; for near-term scalability, optimized bimetallic vanadium-based MXenes offer a more practical balance. Future efforts may also explore anionic doping (e.g., N, B, S) to further tune electronic structure and redox activity.

In summary, to accelerate the development and deployment of vanadium-based MXenes for aqueous supercapacitors, we identify the following key research priorities and practical challenges: (1) Atomic-scale design: Elucidate the precise roles of multi-metal ordering, surface termination, and defect engineering in governing electronic structure and ion transport. (2) Operando characterization: Employ in situ/operando techniques (e.g., XAS, Raman, EQCM) to track dynamic structural and chemical evolution during cycling, particularly in multimetallic and high-entropy systems. (3) Oxidation resilience: Develop surface passivation strategies and synthesis protocols that enhance ambient stability without compromising electrochemical activity. (4) Scalable synthesis: Advance fluorine-free, low-energy etching methods and continuous processing routes (e.g., roll-to-roll) to enable large-scale production with consistent quality. (5) Electrolyte compatibility: Establish systematic electrolyte design rules tailored to the specific redox chemistry and interlayer chemistry of vanadium-based MXenes. (6) Device integration: Demonstrate prototype devices (e.g., flexible micro-supercapacitors, hybrid systems) that leverage the unique properties of vanadium-based MXenes in real-world application scenarios.

## Figures and Tables

**Table 1:** Summary of electrochemical performance of representative vanadium-based MXenes for aqueous supercapacitors.

Materials	Electrolyte	Specific Capacitance	Cycling Stability	Refs.
V_2_CT*_z_*	2 M Zn(CF_3_SO_3_)_2_	54.12 mF cm^−2^ (0.1 mA cm^−2^)	81.48% after 8000 cycles	[29]
V_4_C_3_T*_z_*	1 M H_2_SO_4_	209 F g^−1^ (2 mV s^−1^)	97.23% after 10,000 cycles	[30]
Ti_2_VC_2_T*_z_*	1 M H_2_SO_4_	520 F g^−1^ (2 mV s^−1^)	>90% after 20,000 cycles	[34]
Mo_2_VC_2_T*_z_*	1 M H_2_SO_4_	257.6 F g^−1^ (0.1 A g^−1^)	95.71% after 20,000 cycles	[35]
(Mo_1/3_V_2/3_)_2_CT*_z_*	2 M ZnSO_4_	608.6 F g^−1^ (0.2 A g^−1^)	94.4% after 13,000 cycles	[12]
TiVMoC_2_T*_z_*	1 M H_2_SO_4_	1081.6 F g^−1^ (5 mV s^−1^)	90.8% after 30,000 cycles	[35]

## Data Availability

The data that support the findings of this study are available from the corresponding author upon reasonable request.
